# Ascorbic Acid Ameliorates Cardiac and Hepatic Toxicity Induced by Azithromycin-Etoricoxib Drug Interaction

**DOI:** 10.3390/cimb44060172

**Published:** 2022-05-31

**Authors:** Reham Z. Hamza, Fatima S. Alaryani, Fatma Omara, Mahmoud A. A. Said, Sayed A. Abd El-Aziz, Sawsan M. El-Sheikh

**Affiliations:** 1 Biology Department, Faculty of Sciences, Taif University, P.O. Box 11099, Taif 21944, Saudi Arabia; 2 Biology Department, Faculty of Sciences, University of Jeddah, Jeddah 21959, Saudi Arabia; fsalaryani@uj.edu.sa; 3 Central Hospital of Al-Qenayat, Zagazig 7111214, Egypt; bataomara@gmail.com; 4 Zagazig University Hospital, Zagazig University, Zagazig 44519, Egypt; ph.mahmoud.91@gmail.com; 5 Department of Pharmacology, Faculty of Veterinary Medicine, Zagazig University, Zagazig 44519, Egypt; sayedabdelaziz44@gmail.com (S.A.A.E.-A.); sawsan.ali.elsheikh@gmail.com (S.M.E.-S.)

**Keywords:** histopathology, etoricoxib-azithromycin, ascorbic acid, male albino rats, hepatotoxicity, cardiotoxicity

## Abstract

The complexity of prescribing safe and effective drug therapy is still challenging. Due to the increased number of medications taken by patients, the potential for drug-drug interactions has clinically important consequences. This study focuses on the potential drug-drug interaction between azithromycin and etoricoxib and the possibility of counteracting this adverse reaction by giving ascorbic acid intraperitoneally to male albino rats. Sixty adult male albino rats weighing 150–180 g were used. The rats were allocated into six equal groups. One group was a control, and the others were given azithromycin, etoricoxib, either alone or combination, with one group treated with ascorbic acid and the last group treated with the drug combination and ascorbic acid. Blood samples were collected for measuring AST, ALT, LDH, CK-MB, and troponin alongside antioxidant enzymes and histopathological examination for both liver and heart tissue. The results showed both hepatic and cardiac damage in azithromycin and etoricoxib groups represented by increasing levels of heaptoc enzymes (ALT, AST, LDH, CK-MB, and troponin) with declining antioxidant enzymes and elevation of malondialdehyde and the appearance of hepatic and cardiac toxicities. Upon administration, ascorbic acid ameliorated all the mentioned biochemical parameters. In conclusion, ascorbic acid has great antioxidant capacities and hepatic and cardiac ameliorative effects and can alleviate drug interaction toxicity.

## 1. Introduction

Nowadays, the complexity of prescribing safe and effective drug therapy is still challenging. As the number of medications taken by the individual patient increases, so does the potential for drug-drug interactions that have clinically important consequences [1]. Recent regulatory actions by the US Food and Drug Administration (FDA) remind us of the potential risk of important drug interactions with anti-infective agents [2]. In the early 1990s, patients experienced serious cardiac toxicity after taking antihistaminic drugs in combination with macrolide antibiotics. The inhibition of cytochrome P450 (CYP450) 3A isoenzymes by azithromycin resulting in higher plasma drug concentrations was the major explanation.

Azithromycin is a macrolide antibiotic and the sole member of the azalide subclass. It has broad antimicrobial activity against both anaerobic and aerobic Gram-positive and Gram-negative bacteria. Until recently azithromycin was considered to have minimal cardiac effects compared to the other antibiotics in its class [3] (Minji, 2014). Some data has been reported that azithromycin interacts with etorcoxib (Selective COX-2 inhibitor).

Recently, etoricoxib, a new selective COX-2 inhibitor was developed. It has potent analgesic and anti-inflammatory effects with higher tolerability and fewer gastrointestinal adverse effects than other non-steroidal anti-inflammatory drugs (NSAIDs). Despite its safety on (Gastrointestinal tract) GIT, cardiovascular problems may occur [4]. However, the evaluation of such risks is still difficult, as well as the evaluation of possible adverse effects on the cardiovascular system, since there have been no proper studies on the interaction between etoricoxib and azithromycin.

Ascorbic acid is a widely known vitamin in different kinds of fruits and is available as a supplement. Ascorbic acid plays an important physiological role as a reducing agent and free radical scavenger agent. There is a strong functional relation between antioxidant enzymes, such as GSH, and ascorbic acid, as it was noted that there is a great increase in glutathione levels in the liver and muscle after administration of ascorbic acid [5].

Reactive oxygen species (ROS) are generated as a result of oxygen metabolism. Normally, ROS generated inside the human body is exposed to neutralization by the enzymatic and non-enzymatic antioxidant defense system, including CAT, GSH, and SOD [6] However, upon the exposure of humans and animals to environmental pollutants or drug or chemical toxicity, chemicals elevate dramatically the production of ROS, leading to an imbalance in oxidative status [7].

Guanylate cyclase seemed to be a nonspecific tissue enzyme; however, in light of clinical trials, it became an interesting target for pharmacological intervention. There are several possible options which lead to an elevation in cyclic guanosine monophosphate concentrations. One option involves increasing the concentration of cyclic guanosine monophosphate by the additional direct activation of soluble guanylate cyclase. Treatment is based on the modulation of the guanylate cyclase function. Additionally, most of these agents are very effective in the chronic treatment of patients with heart failure and pulmonary hypertension [8].

To assess the activity of ascorbic acid as cardioprotective and hepatoprotective, several markers have been chosen to be tested in the current research to give an insight into how well the heart and liver is functioning. Additionally, serum ALT, AST, LDH, Ck-MB, troponin, superoxide dismutase (SOD), malondialdehyde (MDA), and glutathione (GSH) levels can all serve as indicators of liver and heart function and oxidative stress [9]. While there have been several previous investigations on the ameliorative capacities of ascorbic acid, no previous research has shed light on both the cardioprotective and hepatoprotective effects of ascorbic acid against drug interactions between azithromycin and etoricoxib. Therefore, the objective of the current study is to evaluate the potential cardiotoxicity and hepatotoxicity that occurs due to the drug-drug interaction between etoricoxib and azithromycin and the possibility of counteracting this adverse effect by giving ascorbic acid intraperitoneally to male albino rats.

## 2. Material and Method

### 2.1. Drugs

Etoricoxib, ascorbic acid 500 mg/mL, azithromycin 100 mg/5 mL, and powder for oral suspension were obtained from Egyphar Co., Obour city, Egypt.

### 2.2. Animals and Experimental Design

**Figure 1:**
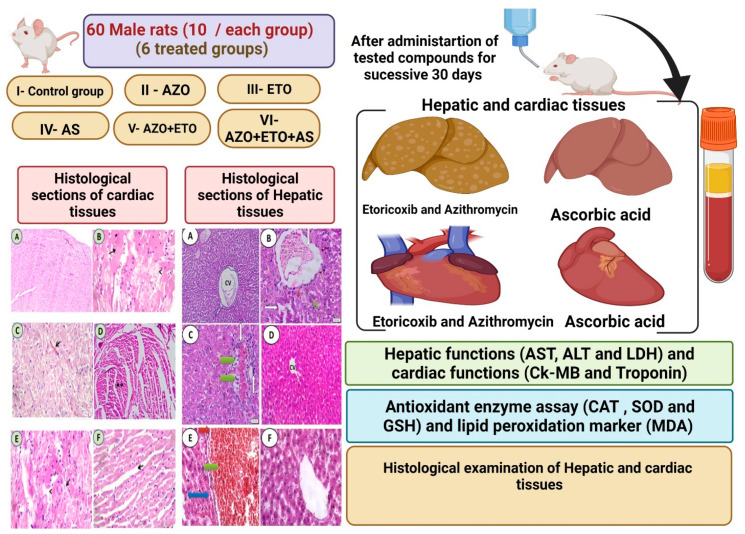
Experimental Protocol.

Sixty adult male albino rats weighing 150–180 gm were used. They were obtained from the Animal Breeding Unit, Faculty of Veterinary Medicine, Zagazig University. The rats were left to acclimatize in a wire cage with natural ventilation and were given a standard pelleted diet and clean tap water ad libitum 2 weeks before the experiment. The male rats were allocated into 6 equal groups, each consisting of 10 male rats. They were treated for 30 successive days as in Table 1 and experimental protocol (Figure 1). The experiment had the ethical approval number (ZU-IACUC/2/F/69/2020).

### 2.3. Blood Collection

Blood samples were taken on days 1 and 14 post treatment from eye plexus as it gives the purest blood. Then, after 30 days post treatment, the rats were sacrificed after light ether anesthesia by ketamine. We made every effort to comply with the strict guidelines to reduce pain and suffering. Then, blood samples were collected on tubes without any anticoagulants and then centrifuged at 5000 r.p.m to obtain serum. Then, we collected the obtained serum in Eppendorf tubes to be used for measuring biochemical liver and heart function parameters.

### 2.4. Liver Enzymes Function Biomarkers

ALT, AST, and LDH were evaluated in serum using available commercial kits (Spinreact, Spain).

### 2.5. Preparation of Hepatic Tissue Homogenates for the Determination of the Redox State

A small portion from hepatic tissues was used to determine the oxidative injury. The hepatic tissues were suddenly immersed in a slightly basic phosphate buffer and centrifuged to obtain the supernatant of hepatic tissues homogenates.

### 2.6. Hepatic Antioxidant Enzymes and Oxidative Stress Marker

Commercial kits were used for determination of antioxidant enzymes and oxidant marker; kits were obtained from Bio-diagnostic, co., Cairo, Egypt. Malondialdehyde, a final lipid peroxidation marker, also known as MDA, was assayed in the liver tissues [12]. Glutathione (GSH) was measured as the reaction was monitored indirectly with the oxidation rate of NADPH at 240 nm for 3 min. The enzyme activity was expressed as nmol/100 mg protein [12] and superoxide dismutase (SOD) [13] was measured by assaying the auto oxidation of pyrogallol at 440 nm for 3 min. Its activity is expressed as U/g. Catalase (CAT) activity was measured by assaying the hydrolysis of H_2_O_2_ and the resulting decrease in absorbance at 240 nm. Its activity is expressed as U/g [14,15] and was determined in the homogenates of the liver in all male rats.

### 2.7. Determination of Heart Biomarkers

Commercial kits and a clinical chemistry automatic analyzer (Hitachi 912, Roche Diagnostic GmbH, Mannheim, Germany) were used for the measurement of serum levels of troponin (Tn I) and creatine kinase (CK-MB).

### 2.8. Histopathological Study

The formalin was used for the fixation of both liver and heart tissues. They were processed in an automated tissue processor. Paraffin sections (4–5 µm) were stained with hematoxylin and eosin. Stained sections were examined for inflammatory reactions, degenerative, necrotic, apoptotic changes, and any other pathological lesions in the examined tissues of experimental rats [16].

### 2.9. Statistical Analysis

The obtained data were analyzed and graphically represented using the statistical package for social science (SPSS, version 16) for obtaining the mean value ± standard error. The results were statistically analyzed by using a one-way ANOVA test. Subsequent multiple comparisons between the different groups were analyzed by Duncan’s multiple comparison tests. Values at *p* < 0.05 were considered significant [17]. 

## 3. Results

### 3.1. Ascorbic Acid Alleviated Liver Injury in Male Rats Exposed to Combination of Etoricoxib and Azithromycin

Etoricoxib and azithromycin administration for 30 successive days afforded a significant increase in the serum activities of ALT, AST, and LDH in male rats. By contrast, the supplementation of ascorbic acid ameliorated hepatic enzymes and LDH in the etoricoxib and azithromycin-treated group in male rats (Table 2 and Table 3).

### 3.2. Ascorbic Acid Alleviated Oxidative Stress in Male Rats Exposed to Combination of Etoricoxib and Azithromycin

Reactive oxygen species levels manifested a marked elevation in the hepatic tissues of either the azithromycin and/or etoricoxib combination in male rats (Table 4). The supplementation of ascorbic acid decreased the hepatic reactive oxygen levels in azithromycin and/or etoricoxib-treated male rats. The levels of hepatic MDA, which is a final marker of lipid peroxidation (Table 4), were significantly elevated (*p* < 0.001) in azithromycin and/or etoricoxib-treated male rats. These structural changes were mainly inversed in the group administered with ascorbic acid.

On the contrary, in male rats exposed to azithromycin and/or etoricoxib, hepatic tissues exhibited significant decreases (*p* < 0.05) in the GSH contents, SOD activity (Table 4) and CAT activities, as compared with those of the control group. Oral supplementation of ascorbic acid elevated the levels of antioxidant enzymes (SOD, CAT, and GSH) in the hepatic tissues of azithromycin and/or etoricoxib-exposed male rats. In addition, ascorbic acid decreased MDA levels in the hepatic tissues of normal rats compared with the azithromycin and/or etoricoxib-treated group.

### 3.3. Ascorbic Acid Alleviated Cardiac Injury in Male Rats Exposed to Combination of Etoricoxib and Azithromycin

The above-mentioned dose of azithromycin and its combination with etoricoxib caused a significant increment in the serum creatine kinase and serum troponin of azithromycin, including in its combination with the etoricoxib-administered group along the entire period of the study. In addition, the serum troponin of azithromycin and its combination with the etoricoxib-administered group increased significantly in contrast to the control group during the entire period of the study. The administration of ascorbic acid combined with the two drugs caused a significant reduction of serum troponin and creatine kinase (*p* < 0.05) along the entire period of the study in comparison with groups receiving the test drugs and their combinations (Table 5 and Table 6).

### 3.4. Histopathological Results

**Figure 2:**
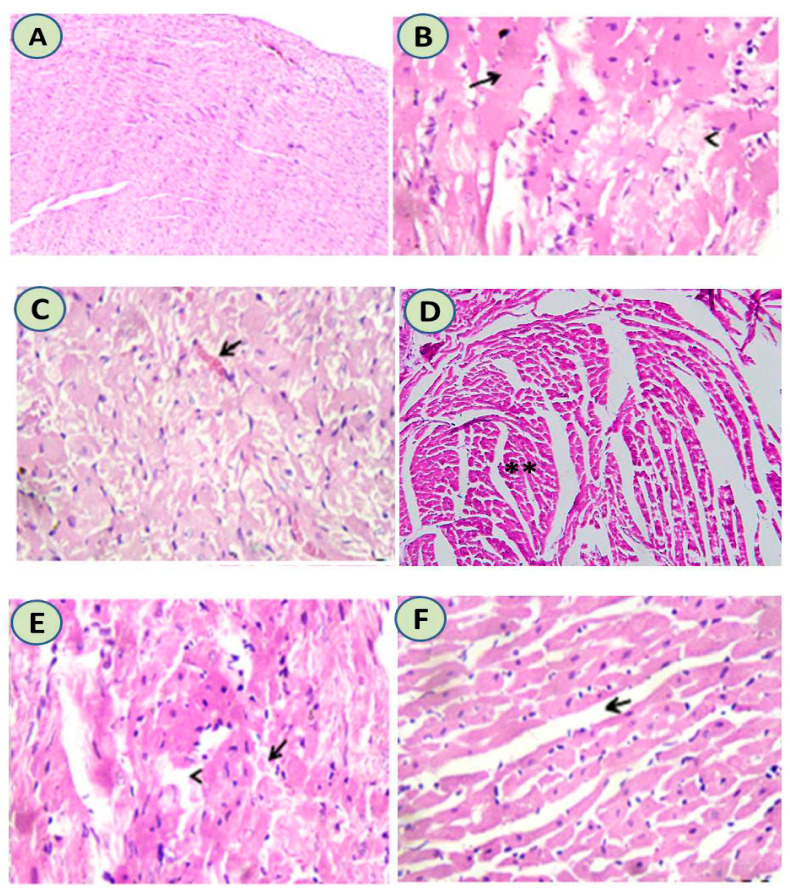
(**A**) The heart tissue of the control group was normal in structure (H&EX200). (**B**) Azithromycin-treated group showed hyaline degeneration associated with pyknotic nuclei and myolysis (H&EX400). (**C**) Etoricoxib-treated group showed intramuscular edema and congested capillaries, and partial necrotic changes were evident in the myocardium. (H&EX400) (**D**) Ascorbic acid-treated group showed normal cardiac tissues with normal intramuscular muscles (**) with normal sized nuclei (H&EX200). (**E**) Etoricoxib-azithromycin treated group showed hyalinized or necrotic myocardial muscle fibers alongside a few scattered lymphocytes among necrotic muscle fiber (H&EX400). (**F**) Upon administration of ascorbic acid with azithromycin and etoricoxib, it was shown that the majority of muscle fibers nearly returned to their normal state with mild edema and few scattered lymphocytes (H&EX400).

Histological examination of cardiac tissues showed normal heart tissues in the control group. Meanwhile, administration of either azithromycin or etoricoxib or their combination induced hyaline degeneration associated with pyknotic nuclei and myolysis. Intramuscular edema, congested capillaries, and partial necrotic changes were evident in the myocardium. Upon administration of ascorbic acid with the azithromycin and etoricoxib combination, the majority of muscle fibers nearly returned to their normal state with few scattered lymphocytes, as shown in Figure 2.

**Figure 3:**
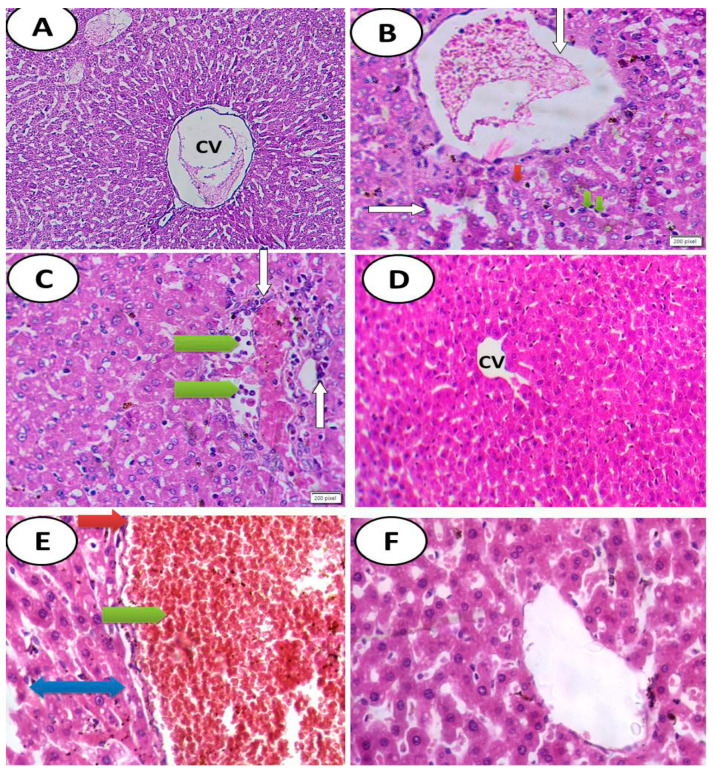
(**A**) Cross section of hepatic tissues of control group showing normal hepatic structure with normal hepatocytes (H&EX100). (**B**) Photomicrograph of cross section of experimental rat liver after administration of azithromycin, showing marked toxicity in the form of hypertrophy of hepatocytes with appearance of binucleated hepatocytes (green arrow), increased eosinophilia, granular cytoplasm and vesicular nuclei (red arrow), with damage of the normal architecture of the liver, the portal vein dilated and congested (white arrow), dilatation of some blood sinusoids, infiltration by inflammatory cells, and appearance of apoptotic bodies (H&EX400). (**C**) Photomicrograph of cross section of experimental rat liver after administration of etoricoxib drug showing marked toxicity in the form of hypertrophy of hepatocytes with appearance of binucleated hepatocytes and increased eosinophilia, granular cytoplasm and vesicular nuclei (green arrow), with damage of the normal architecture of the liver, the portal vein dilated and congested (white arrow), new bile duct formation, ductular reaction at the periphery of the portal tract, and infiltration of the portal tract by mononuclear inflammatory cells. (**D**) Cross section of experimental rat liver after administration of ascorbic acid showing normal hepatic structure with normal central vein (CV) and normal hepatocytes (H&EX200). (**E**) Photomicrograph of cross section of experimental rat liver after administration of combination of azithromycin and etoricoxib drugs showing severe toxicity in the form of hypertrophy of hepatocytes with granular eosinophilic cytoplasm and vesicular nuclei and appearance of some binucleated cells, marked dilatation of the central vein and severe hemorrhage (green arrow), perivenular fibrosis (blue two-headed arrow), focal areas of necrosis, and ballooning degeneration in some hepatocytes (red arrow) (H&EX400). (**F**) Photomicrograph of cross section of experimental rat liver after administration of combination of azithromycin and etoricoxib drugs combined with ascorbic acid showing high alleviation of drug toxicity with mild toxicity in the form of hypertrophy of hepatocytes with granular eosinophilic cytoplasm and vesicular nuclei and appearance of some binucleated cells with slightly dilated central vein lined by endothelial cells (H&EX400).

Histological sections showed normal hepatic structure with normal hepatocytes in both control and ascorbic acid groups. Meanwhile, the appearance of hepatic damage in groups treated with either azithromycin and/or etoricoxib or their combination was represented by the hypertrophy of hepatocytes, increased eosinophilia, granular cytoplasm and vesicular nuclei with marked dilatation of the central vein and severe hemorrhage. By contrast, there was the appearance of high amelioration in groups treated with combination of drugs and followed by ascorbic acid, as shown in Figure 3.

## 4. Discussion

This study examined the impact of 14 consecutive days of an azithromycin course and azithromycin combined with etoricoxib. The purpose of this study aimed to investigate the possible interaction between azithromycin and etoricoxib causing abnormalities in heart functions and myocardial tissues and how to avoid them by giving ascorbic acid.

In our study, the myolysis of the myocardial tissue of rats receiving azithromycin and the myocardial necrosis of the heart tissue of rats receiving azithromycin with etoricoxib was supported by the significant elevations that occurred in the serum troponin and creatine kinase activities. Our results agreed with Alti et al. [18]; he found that 5-day azithromycin (10 mg/kg) was associated with cardiotoxicity in rats. The results of this study were compatible with El-Shitany and El-Desoky [19], who concluded that azithromycin 10 mg/kg given to rats caused a marked atrophy of cardiac muscle fibers with increased tissue spaces.

Concerning the oral administration of etoricoxib given to rats, some muscle fibers were partially necrotized. Etoricoxib, a selective inhibitor of COX-II and anti-inflammatory drug is less likely to cause gastrointestinal problems [20].

A possible problem due to etoricoxib administration is the increased risk of cardiovascular disorders, e.g., heart failure and myocardial infarction [21]. Kothapalli et al. [22] underlined our data, stating that COX-2 is expressed in various normal tissues where its expression may be important for physiological functions such as cardioprotection; therefore, its inhibition could result in serious cardiovascular events.

Noticeably, the dose of azithromycin combined with etoricoxib caused an increase in the activities of troponin and CK-MB in a significant manner, associated with myocardial necrosis. Generally, it is known azithromycin is susceptible to interaction with certain drugs (Maisch et al.) [23]. No specific studies have been conducted on the possibility of cardiotoxicity induced by azithromycin with etoricoxib. One major reason for this is that some data has reported that azithromycin inhibits the metabolism of etoricoxib, thus increasing the risk of cardiotoxicity.

**Figure 4:**
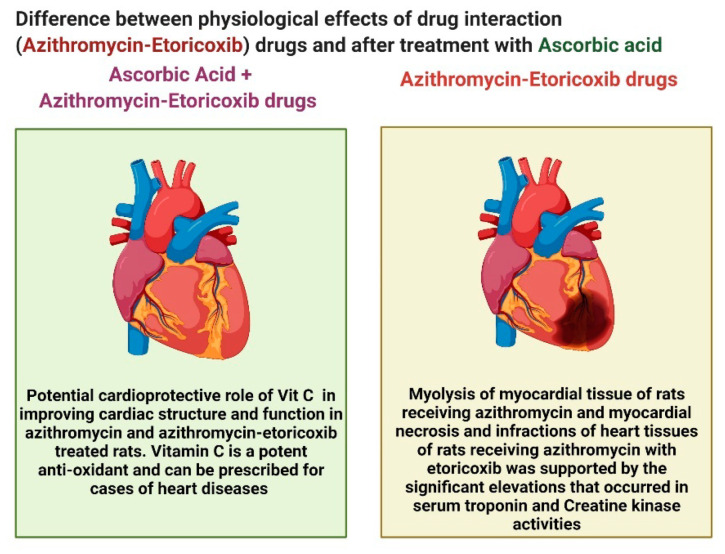
Comparison between drug interactions (azithromycin and etoricoxib) and after ascorbic acid.

Thus, it is clearly demonstrated that the combination of azithromycin and etoricoxib induced heart infractions, necrosis, and infraction of cardiac muscles, which is contrary to the addition of ascorbic acid, which induced great improvement in cardiac tissues and alleviated the cardiac toxicity induced due to the interaction between the two antibiotics, as clearly illustrated in Figure 4.

Excess vasoconstriction is an important contributing factor to the pathogenesis of essential hypertension. Direct vasodilators target the smooth muscle cells to decrease vascular tone and blood pressure. Azithromycin is in the category of macrolides, which induce phase III-like contractions in the human gastrointestinal tract during the interdigestive state that stimulates motor activity in human smooth muscle receptors [24]. Azithromycin has also been found to promote gastrointestinal activities and thus there is a direct relationship between the drug interaction and muscular contractions, which may confirm the current findings.

**Figure 5:**
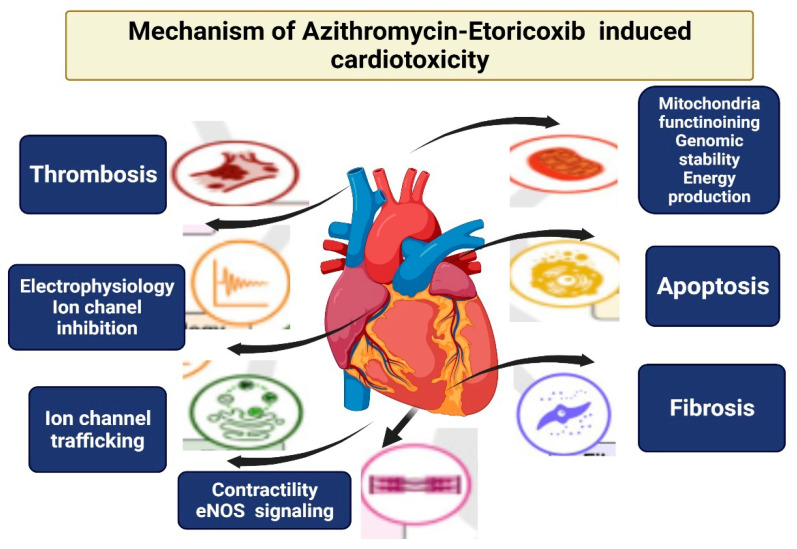
Mechanism of action of drug interactions causing cardiotoxicity.

The general results from the simultaneous interruption of myocardial functions and viabilities could confirm the cardiotoxicity induced by both azithromycin and etoricoxib. The adverse drug effects were the disruption of electrophysiology, contractility, mitochondrial toxicity, growth factor, and cytokine regulation. These mechanisms of drug cardiotoxicity are shown in Figure 5. The mechanism underlying adverse cardiac series often exert their action through complex cellular signaling pathways. The main signaling pathways underlie the drug interactions’ cardiotoxicity.

Azithromycin is a widely used antibiotic in the treatment protocol of COVID-19 for its antiviral and immunomodulatory effects. Previously, rat models showed that azithromycin produces high free radicals, and thus induces excessive oxidative stress, inflammation, and infraction of myocardial tissue [25]. Thus, azithromycin induces cardiotoxicity. These findings are in complete agreement with the current findings.

The present study provides evidence for the potential cardioprotective role of ascorbic acid (40 mg/kg) in improving cardiac structure and function in azithromycin and azithromycin-etoricoxib-treated rats. Ascorbic acid is a potent antioxidant and can be prescribed for cases of heart diseases, e.g., coronary heart diseases and myocardial infarction (Yogeeta et al.) [26]. Our data were well-matched with Swamy et al. [27], who have reported that ascorbic acid 20 mg p.o restored heart function parameters and structures in rats treated with doxorubicin.

Thus, the importance of this study comes from its concept of using ascorbic acid in cases of COVID-19, which could alleviate the oxidative stress induced by antibiotics that are used to treat the SARS-CoV-2 side effects on different organs. This is a significant confirmation of the potent antioxidant capacities of ascorbic acid against oxidative injury induced by antibiotics during the COVID-19 pandemic. Thus, the use of antioxidants is of great importance in alleviating oxidative stress injuries in different organs, as confirmed previously [28,29,30,31,32].

The current findings revealed that ALT, AST, and LDH were increased significantly in groups treated with the combination of both azithromycin and etoricoxib and induced liver injury in the histological examination. These increased levels of AST, ALT, and LDH may be indicative of heptocellular leakage, loss of hepatocyte functional integrity, and infiltration through the hepatic cellular membrane. The hepatocellular leakage caused these enzymes to leak out of the liver into the blood circulation. Serum ALT levels are considered a more specific hepatic marker in the hepatic tissues and thus evaluate the extent of liver injury. Additionally, elevated serum AST levels are indicative of increased hepatic pressure [33,34].

These findings confirmed that ascorbic acid attenuated the hepatic parenchymal necrosis induced by the azithromycin-etoricoxib interaction in male rats for a small period of time (only two weeks). This hepatic damage was higher in the current study, by induction for a long period of 30 days, and also confirmed the potent antioxidant role of ascorbic acid in the alleviation of oxidative stress induced by ascorbic acid [33].

The current findings are parallel with Yin et al. [34], who demonstrated that ascorbic acid is a potent antioxidant with scavenging free radicals and thus improves general health and also affords strong synergetic effects with other chemicals and vitamins, which elevate its activity, enhance the immune system, and can alleviate oxidative damage and reduce the excessive production of free radicals. 

Previous studies from Qinna et al. [35] confirmed that ascorbic acid can enhance the restoration of normal hepatic functions. This significantly confirms the current findings as we proved both the hepatoprotective and cardioprotective effects of ascorbic acid after azithromycin and etoricoxib-treatment, compared to treatment with azithromycin and etoricoxib alone.

These finding are also in complete accordance with Zoheir et al. [36], who also reported that the use of multiple antioxidant therapies can restore normal hepatocyte function.

## 5. Conclusions

It is well known that etoricoxib and azithromycin have cardiovascular and hepatic adverse effects, although no specific study has been performed on their interaction. From this study, it is concluded that ascorbic acid counteracts heart and liver functions and the histological abnormalities caused by the azithromycin-etoricoxib interaction.

## Figures and Tables

**Table 1:** Compounds of study.

Experimental Animals	Dose
I. Control group	Were given 1 mL distilled water.
II. Treated with azithromycin	Azithromycin (45 mg/kg) [9] for 30 consecutive days.
III. Treated with etoricoxib	Etoricoxib (1 mg/kg) [10] for 30 consecutive days.
IV. Treated with ascorbic acid	Ascorbic acid (40 mg/kg) (i.p) injected for 30 consecutive days [11].
V. Treated with etoricoxib and azithromycin	Etoricoxib and azithromycin were orally administered every day for 30 consecutive days.
VI. Treated with etoricoxib, azithromycin, and ascorbic acid	Etoricoxib, azithromycin combined with ascorbic acid (i.p) were given for 30 consecutive days.

**Table 2:** Effects of ascorbic acid on hepatic enzymes in male rats after 30 days of administration of either azithromycin or etoricoxib or their combinations.

	ALT (U/L)			AST (U/L)		
	1st Day	14th Day	30th Day	1st Day	14th Day	30th Day
Control Azithromycin (AZO)	13.02 ± 0.87 ^d^	14.02 ± 1.02 ^d^	13.02 ± 1.02 ^e^	14.36 ± 1.69 ^d^	13.69 ± 2.02 ^f^	14.01 ± 1.69 ^ef^
	3.02 ± 1.87 ^d^	63.02 ± 2.87 ^c^**	141.02 ± 5.87 ^b^***	23.02 ± 0.87 ^b^**	53.02 ± 0.87 ^b^**	83.02 ± 0.87 ^c^**
Etoricoxib (ETO)	34.25 ± 2.36 ^a^**	88.65 ± 2.65 ^b^**	120.36 ± 2.65 ^c^***	20.36 ± 1.69 ^c^	44.36 ± 1.69 ^c^**	87.65 ± 2.05 ^bc^**
Ascorbic acid (AS)	13.25 ± 1.25 ^d^	12.69 ± 1.39 ^d^	12.58 ± 1.63 ^e^	15.99 ± 1.85 ^d^	14.25 ± 1.36 ^ef^	12.69 ± 1.69 ^f^
AZO + ETO	28.36 ± 3.21 ^b^*	98.69 ± 2.01 ^ab^**	189.25 ± 1.69 ^a^***	29.67 ± 2.69 ^a^*	78.25 ± 2.01 ^a^***	110.98 ± 2.69 ^a^***
AZO + ETO + AS	21.36 ± 1.25 ^c^*	67.36 ± 2.01 ^c^**	47.02 ± 2.36 ^d^**	21.36 ± 2.03 ^c^*	35.35 ± 1.47 ^d^**	69.15 ± 1.69 ^d^**

Results are expressed as mean ± SE. Mean values with different letters in the same row show significance at *p* ≤ 0.05, where the highest mean value has the symbol ^a^ and ^b^,^c^,^d^,^f^: For significance: * for *p* < 0.05, or ** for *p* < 0.01, or *** for *p* < 0.001. AZO: Azithromycin, ETO: Etoricoxib, AS: Ascorbic acid, ALT: Alanine aminotransferase, AST: Aspartate aminotransferase, LDH: Lactate dehydrogenase enzyme.

**Table 3:** Effects of ascorbic acid on lactate dehydrogenase enzyme (LDH) in male rats after 30 successive days of administration of either azithromycin or etoricoxib or their combinations.

Groups	LDH (U/L)		
	1st Day	14th Day	30th Day
Control	225.95 ± 2.69 ^d^	234.02 ± 4.25 ^c^	230.58 ± 2.98 ^de^
Azithromycin (AZO)	478.69 ± 3.69 ^b^**	499.55 ± 4.02 ^b^	680.69 ± 7.58 ^c^***
Etoricoxib (ETO)	455.36 ± 5.25 ^b^***	469.82 ± 3.25 ^b^	785.22 ± 2.99 ^b^***
Ascorbic acid (AS)	157.69 ± 2.02 ^e^*	148.52 ± 3.69 ^d^**	145.25 ± 3.25 ^f^**
AZO + ETO	620.36 ± 3.65 ^a^***	785.29 ± 5.69 ^a^***	920.39 ± 5.41 ^a^***
AZO + ETO + AS	245.69 ± 4.25 ^cd^*	241.39 ± 2.69 ^c^*	220.39 ± 4.25 ^e^*

Results are expressed as mean ± SE. Mean values with different letters in the same row show significance at *p* ≤ 0.05, where the highest mean value has the symbol ^a^ and ^b^,^c^: For significance: * for *p* < 0.05, or ** for *p* < 0.01, or *** for *p* < 0.001. AZO: Azithromycin, ETO: Etoricoxib, AS: Ascorbic acid, LDH: Lactate dehydrogenase enzyme.

**Table 4:** Effects of ascorbic acid on oxidative stress enzymes and oxidative damage markers in hepatic tissues in male rats after administration of either azithromycin or etoricoxib or their combinations after 30 days post administration.

Parameters	Control	AZO	ETO	AS	AZO + ETO	AZO + ETO + AS
CAT (U/g)	11.02 ± 0.26 ^a^	2.42 ± 0.16 ^c^***	1.57 ± 0.76 ^c^***	11.98 ± 2.25	1.98 ± 0.87 ***	8.52 ± 1.69 **
SOD (U/g)	16.20 ± 0.26 ^a^	10.07 ± 0.45 ^c^***	8.17 ± 1.45 ^c^***	17.02 ± 2.25	7.05 ± 1.58 ***	13.82 ± 0.34 ^a^**
GSH (U/g)	15.78 ± 2.38 ^b^	8.10 ± 0.88 ^c^**	6.80 ± 1.88 ^c^**	16.25 ± 1.58	5.25 ± 0.58 **	12.12 ± 0.33 ^ab^**
MDA (µg/mg)	3.01 ± 0.15 ^cd^	14.01 ± 1.30 ^a^***	15.41 ± 1.80 ^a^	2.01 ± 0.58	30.25 ± 2.02 ***	7.05 ± 0.68 ^d^**

Results are expressed as mean ± SE. Mean values with different letters in the same row show significance at *p* ≤ 0.05, where the highest mean value has the symbol ^a^ and ^b^,^c^: For significance: ** for *p* < 0.01, or *** for *p* < 0.001. AZO: Azithromycin, ETO: Etoricoxib, AS: Ascorbic acid, CAT: Catalase, SOD: Superoxide dismutase, GSH: Glutathione, MDA: Malondialdehyde.

**Table 5:** Effects of ascorbic acid on heart function marker (CK-MB) in male rats after 30 days of administration of either azithromycin or etoricoxib or their combinations.

Groups	CK-MB (U/L)		
	1st Day	14th Day	30th Day
Control	71.02 ± 6.25 ^e^	123.66 ± 7.18 ^e^	151.02 ± 5.65 ^e^
Azithromycin	73.83 ± 6.49 ^de^	171.5 ± 6.29 ^d^**	471 ± 6.14 ^b^***
Etoricoxib	160.66 ± 5.4 ^a^***	277.66 ± 9.55 ^bc^***	412 ± 5.85 ^c^***
Ascorbic acid	70.02 ± 4.58 ^e^	123.66 ± 7.18 ^e^	157.33 ± 5.64 ^e^
Azithromycin + Etoricoxib	106.66 ± 3.004 ^b^***	311.66 ± 6.78 ^a^***	575 ± 9.15 ^a^***
Azithromycin + Etoricoxib + Ascorbic acid	88.66 ± 5.36 ^cd^	230.66 ± 1.74 ^c^	223 ± 11.02 ^d^

Results are expressed as mean ± SE. Mean values with different letters in the same row show significance at *p* ≤ 0.05, where the highest mean value has the symbol ^a^ and ^b^,^c^: For significance: ** for *p* < 0.01, or *** for *p* < 0.001. AZO: Azithromycin, ETO: Etoricoxib, AS: Ascorbic acid, CAT: Catalase, SOD: Superoxide dismutase, GSH: Glutathione, MDA: Malondialdehyde.

**Table 6:** Effects of ascorbic acid on heart function marker (troponin) in male rats after 30 days of administration of either azithromycin or etoricoxib or their combinations.

Groups	Troponin (U/L)		
	1st Day	14th Day	30th Day
Control	0.05 ± 0.03 ^e^	0.15 ± 0.03 ^e^	0.25 ± 0.03 ^e^
Azithromycin	5.01 ± 0.58 ^b^	7.01 ± 0.58 ^b^*	9.01 ± 0.58 ^b^
Etoricoxib	4.98 ± 0.57 ^c^**	8.98 ± 0.57 ^c^**	10.98 ± 0.57 ^c^**
Ascorbic acid	0.04 ± 0.003 ^e^	0.14 ± 0.003 ^e^	0.30 ± 0.003 ^e^
Azithromycin + Etoricoxib	7.04 ± 1.3 ^ab^**	9.04 ± 1.3 ^ab^**	14.04 ± 1.3 ^ab^**
Azithromycin + Etoricoxib + Ascorbic acid	2.16 ± 0.82 ^d^	3.16 ± 0.82 ^d^	4.16 ± 0.82 ^d^

Results are expressed as mean ± SE. Mean values with different letters in the same row show significance at *p* ≤ 0.05, where the highest mean value has the symbol ^a^ and ^b^,^c^: For significance: * for *p* < 0.05, or ** for *p* < 0.01. AZO: Azithromycin, ETO: Etoricoxib, AS: Ascorbic acid, CAT: Catalase, SOD: Superoxide dismutase, GSH: Glutathione, MDA: Malondialdehyde.

## Data Availability

Data generated or analyzed during this study are included in this manuscript.

## References

[1] Coleman J.J., Pontefract S.K. (2016). Adverse drug reactions. Clin. Med..

[2] Spina E., Barbieri M.A., Cicala G., Leon J. (2020). Clinically Relevant Interactions between Atypical Antipsychotics and An-ti-Infective Agents. Pharmaceuticals.

[3] Kim M., Welch T. (2014). Update on azithromycin and cardiac side effects. Southwest Respir. Crit. Care Chron..

[4] Baracho N.C.D.V., Guizelli G.P., Carmello B.L., Sanches D.D.S., Silva F.M.C., Reis J.M.D., Brito J.D. (2009). Cardiovascular and hematologic effects produced by chronic treatment with etoricoxib in normotensive rats. Acta Cirúrgica Bras..

[5] Abdulrazzaq A.M., Badr M., Gammoh O., Abu Khalil A.A., Ghanim B.Y., Alhussainy T.M., Qinna N.A. (2019). Hepatoprotective actions of ascorbic acid, alpha lipoic acid and silymarin or their combination against acetaminophen-induced hepatotoxicity in rats. Medicina.

[6] Hamza R.Z., EL-Megharbel S.M., Altalhi T., Gobouri A.A., Alrogi A.A. (2020). Hypolipidemic and hepatoprotective synergistic effects of selenium nanoparticles and vitamin. E against acrylamide-induced hepatic alterations in male albino mice. Appl. Organomet. Chem..

[7] Abuelzahab H., Hamza R., Montaser M., El-Mahdi M.M., Al-Harthi W.A. (2019). Antioxidant, antiapoptotic, antigenotoxic, and hepatic ameliorative effects of L-carnitine and selenium on cadmium-induced hepatotoxicity and alterations in liver cell structure in male mice. Ecotoxicol. Environ. Saf..

[8] Grześk G., Nowaczyk A. (2021). Current Modulation of Guanylate Cyclase Pathway Activity—Mechanism and Clinical Implications. Molecules.

[9] El-Sayed M.G.A., Kandiel M.M.M., Ebied D.D.I.A. (2017). Changes in reproductive organs, semen characteristics, and in-tra-testicular oxidative stress in adult male rats caused by azithromycin. Int. J. Pharmacol. Toxicol..

[10] Moraes B.M., Amaral B.C.D., Morimoto M.S.S., Vieira L.G.C., Perazzo F.F., Carvalho J.C.T. (2007). Anti-inflammatory and analgesic actions of etoricoxib (an NSAID) combined with misoprostol. Inflammopharmacology.

[11] Ebuehi O.A.T., Ogedegbe R.A., Ebuehi O.M. (2012). Oral Administration of Vitamin C and Vitamin E amelioratesLead-induced Hepatotoxicity and Oxidative Stress in the Rat Brain. Niger. Q. J. Hosp. Med..

[12] Uchiyama M., Mihara M. (1978). Determination of malonaldehyde precursor in tissues by thiobarbituric acid test. Anal. Biochem..

[13] Ellman G.L. (1959). Tissue sulfhydryl groups. Arch. Biochem. Biophys..

[14] Marklund S., Marklund G. (1974). Involvement of the Superoxide Anion Radical in the Autoxidation of Pyrogallol and a Convenient Assay for Superoxide Dismutase. Eur. J. Biochem..

[15] Sinha A.K.J.A.B. (1972). Colorimetric assay of catalase. Anal. Biochem..

[16] Suvarna K.S., Layton C., Bancroft J.D. (2013). Bancroft’s Theory and Practice of Histological Techniques.

[17] Armitage P., Berry G., Matthews J.N.S. (2008). Statistical Methods in Medical Research.

[18] Atli O., Ilgin S., Altuntas H., Burukoglu D. (2015). Evaluation of azithromycin induced cardiotoxicity in rats. Int. J. Clin. Exp. Med..

[19] El-Shitany N.A., El-Desoky K. (2016). Protective Effects of Carvedilol and Vitamin C against Azithromycin-Induced Cardiotoxicity in Rats via Decreasing ROS, IL1-β, and TNF-αProduction and Inhibiting NF-κB and Caspase-3 Expression. Oxidative Med. Cell. Longev..

[20] Hunt R.H., Harper S., Watson D.J., Yu C., Quan H., Lee M., Evans J.K., Oxenius B. (2003). The gastrointestinal safety of the COX-2 selective inhibitor etoricoxib assessed by both endoscopy and analysis of upper gastrointestinal events. Am. J. Gastroenterol..

[21] Cannon C.P., Curtis S.P., FitzGerald G.A., Krum H., Kaur A., Bolognese J.A., Reicin A.S., Bombardier C., Weinblatt M.E., Van Der Heijde D. (2006). Cardiovascular outcomes with etoricoxib and diclofenac in patients with osteoarthritis and rheumatoid arthritis in the Multinational Etoricoxib and Diclofenac Arthritis Long-term (MEDAL) programme: A randomised comparison. Lancet.

[22] Kothapalli D., Fuki I., Ali K., Stewart S.A., Zhao L., Yahil R., Kwiatkowski D., Hawthorne E.A., FitzGerald G.A., Phillips M.C. (2004). Antimitogenic effects of HDL and APOE mediated by Cox-2-dependent IP activation. J. Clin. Investig..

[23] Maisch N.M., Kochupurackal J.G., Sin J. (2013). Azithromycin and the Risk of Cardiovascular Complications. J. Pharm. Pr..

[24] Lenz K.D., Klosterman K.E., Mukundan H., Kubicek-Sutherland J.Z. (2021). Macrolides: From toxins to therapeutics. Toxins.

[25] Mansour B.S., Salem N.A., Kader G.A., Abdel-Alrahman G., Mahmoud O.M. (2021). Protective effect of Rosuvastatin on Azithromycin induced cardiotoxicity in a rat model. Life Sci..

[26] Yogeeta S.K., Gnanapragasam A., Kumar S.S., Subhashini R., Sathivel A., Devaki T. (2006). Synergistic interactions of Ferulic acid with Ascorbic acid: Its cardioprotective role during isoproterenol induced myocardial infarction in rats. Mol. Cell. Biochem..

[27] Swamy A.V., Wangikar U., Koti B.C., Thippeswamy A.H.M., Ronad P.M., Manjula D.V. (2011). Cardioprotective effect of ascorbic acid on doxorubicin-induced myocardial toxicity in rats. Indian J. Pharmacol..

[28] Omara F., Aziz S.A., El-Sheikh S.M., Said M.A.A. (2021). Ascorbic acid attenuated the hepatic parenchymal necrosis induced by azithromycin-etoricoxib interaction in rats. J. Anim. Health Prod..

[29] Hamza R.Z., Sheshah Z.A., Suleman R.H., Al-Juaid N.F., Hamed N.A., Al-Juaid M.A. (2022). Efficacy of some antibiotics and some metal complexes (Nano-formula) that could increase their effectiveness during COVID-19. Int. J. Biol. Pharm. Sci. Arch..

[30] El-Megharbel S.M., Al-Thubaiti E.H., Qahl S.H., Al-Eisa R.A., Hamza R.Z. (2022). Synthesis and Spectroscopic Characterization of Dapagliflozin/Zn (II), Cr (III) and Se (IV) Novel Complexes That Ameliorate Hepatic Damage, Hyperglycemia and Oxidative Injury Induced by Streptozotocin-Induced Diabetic Male Rats and Their Antibacterial Activity. Crystals.

[31] Hamza R.Z., Al-Eisa R.A., El-Shenawy N.S. (2022). Possible Ameliorative Effects of the Royal Jelly on Hepatotoxicity and Oxidative Stress Induced by Molybdenum Nanoparticles and/or Cadmium Chloride in Male Rats. Biology.

[32] El-Megharbel S.M., Al-Baqami N.M., Al-Thubaiti E.H., Qahl S.H., Albogami B., Hamza R.Z. (2022). Antidiabetic Drug Sitagliptin with Divalent Transition Metals Manganese and Cobalt: Synthesis, Structure, Characterization Antibacterial and Antioxidative Effects in Liver Tissues. Curr. Issues Mol. Biol..

[33] Sabiu S., Sunmonu T.O., Ajani E.O., Ajiboye T.O. (2015). Combined administration of silymarin and vitamin C stalls acetaminophenmediated hepatic oxidative insults in Wistar rats. Rev. Bras. Farm..

[34] Yin X., Chen K., Cheng H., Chen X., Feng S., Song Y., Liang L. (2022). Chemical Stability of Ascorbic Acid Integrated into Commercial Products: A Review on Bioactivity and Delivery Technology. Antioxidants.

[35] Qinna N.A., Ghanim B.Y. (2018). Chemical induction of hepatic apoptosis in rodents. J. Appl. Toxicol..

[36] Zoheir K., Amara A.A., Ahmad S.F., Mohammad M.A., Ashour A., Harisa G.I., Abd-Allah A.R. (2014). Study of the therapeutic effects of Lactobacillus and α-lipoic acid against dimethylnitrosamine-induced liver fibrosis in rats. J. Genet. Eng. Biotechnol..

